# Micro-Raman spectroscopy study of blood samples from myocardial infarction patients

**DOI:** 10.1007/s10103-022-03604-1

**Published:** 2022-07-12

**Authors:** Reena V. John, Tom Devasia, Mithun N., Jijo Lukose, Santhosh Chidangil

**Affiliations:** 1 Centre of Excellence for Biophotonics, Department of Atomic and Molecular Physics, Manipal Academy of Higher Education, Manipal, Karnataka 576104 India; 2 Department of Cardiology, Kasturba Medical College, Manipal, Manipal Academy of Higher Education, Manipal, Karnataka 576104 India

**Keywords:** Optical diagnostics, Laser Raman spectroscopy, Myocardial infarction, Inflammation, BH4

## Abstract

Acute myocardial infarction (MI) is found to be a major causative factor for global mortality and morbidity. This situation demands necessity of developing efficient and rapid diagnostic tools to detect acute MI. Raman spectroscopy is a non-destructive optical diagnostic technique, which has high potential in probing biochemical changes in clinical samples during initiation and progress of diseases. In this work, blood was taken as the sample to examine inflammation in acute MI patients using Raman spectroscopy. Ratio of Raman peak intensities that corresponds to phenylalanine (1000 cm^−1^) and tyrosine (825 cm^−1^) can facilitate indirect information about tetrahydrobiopterin (BH4) availability, which can indicate inflammatory status in patients. This ratio obtained was higher for MI patients in comparison with control subjects. The decrease in phenylalanine and tyrosine ratio (Phe-Tyr ratio) is attributed to the prognosis of standard of care (medications like antiplatelets including aspirin, statin and revascularisation) leading to inflammation reduction. Phe-Tyr ratio estimated from the Raman spectra of blood can be exploited as a reliable method to probe inflammation due to MI. The method is highly objective, require only microliters of sample and minimal sample preparation, signifying its clinical utility.

## Introduction

Cardiovascular diseases (CVDs) such as myocardial infarction (MI), coronary artery disease (CAD) and heart failure (HF) are major contributors to global mortality rate. Large number of population is at the risk of developing MI, due to unhealthy dietary habits, smoking, diabetes, genetic predisposition, hypertension, lack of physical activity etc. [1]. CVDs can present at any age and across all demographic ranges. According to the World Health Organization (WHO), CVD is the No.1 “Killer” disease in all the nations across the world [2]. Evolution of atherosclerotic plaques in the coronary arteries—termed coronary artery disease (CAD)—can eventually result in plaque rupture and thrombus formation leading to acute myocardial infarction (AMI). Preliminary diagnosis of MI is performed by monitoring electrocardiographic changes, elevated levels of cardiac biomarkers (Troponin T, myoglobin etc.) [3, 4]. Intense research activities are going on to identify newer biomarkers for early diagnosis of acute myocardial infarction. Early diagnosis of acute MI can help to initiate immediate medical attention and save severe damage to myocardium which will otherwise lead to heart failure. Thus, there is an urgent requirement of technologies for rapid diagnosis of acute MI for ensuring public healthcare.

MI is caused by the atherosclerotic plaque rupture in coronary arteries, as mentioned earlier [3–5]. The plaque has a fibrous cap and fatty lipid core which expands in size with time. The fibrous cap acts as a barrier between the lipid core and blood stream. The rupture of fibrous cap leads to direct contact of blood stream with lipid core, which can give rise to platelet aggregation and thrombus formation which in turn presents as acute MI [6–8]. The blood monocyte level will increase in an event of MI, which accumulates over the thrombosed inflammed plaque lesion, instigating an inflammatory process [9]. Identifying plaque inflammation markers can help to diagnose early MI and myocardial injury. The C-reactive protein (CRP) is one of the most common inflammatory biomarkers amongst the many proteins released in the blood stream. It is well established that patients with elevated level of CRP are prone to develop MI and other cardiovascular diseases [10, 11]. The stimulation of the GTP cyclohydrolase I (GCH) by the monocyte-derived proinflammatory markers alter the synthesis of 5,6,7,8 -tetrahydrobiopterin (BH4), which reduces the conversion of phenylalanine to tyrosine, are well known aromatic amino acids. Phenylalanine is an indispensable amino acid, whereas tyrosine is the semi-indispensable amino acid, because, tyrosine is synthesized from phenylalanine through phenylalanine hydroxylase (PAH) enzyme. The source of these two amino acids in human body other than PAH activity comes through nutritional intake [12–14].

Optical techniques are getting acceptance for medical applications these days in which Raman spectroscopy is a highly non-destructive and sensitive technique based on inelastic scattering of light generated after the interaction of electromagnetic radiation with the sample of interest. Moreover, this label-free technique is highly beneficial for analyzing biological samples since it requires less sample volume with minimal processing time, and not limited by the influence of water solvent [15, 16]. This technique has been found effective in the quantification of bioanalytes such as glucose, total protein, cholesterol, urea, triglycerides, albumin, bilirubin and hemoglobin in blood samples. Quantification of these bioanalytes is highly important for analytical application in medical diagnosis [17]. Application of Raman spectroscopy techniques for cardiovascular diseases is not well documented. There is a report to verify the quality of metal stents for endothelial cell growth in human coronary artery [18]. MI patients have been found with various pathophysiological, biochemical and humoral alterations, with respect to healthy individuals. Higher concentration of troponin in plasma is considered to be the gold standard marker for MI. Similarly, blood samples from cardiac patients have been identified to have raised levels of various biomarkers (BNP, troponin, myoglobin, CK, LDH etc.). Myocardial cell death causes the release of many proteins from injured myocytes into the circulating blood [19]. This demands the necessity of blood samples to be explored as reliable clinical sample for the diagnosis of cardiac disorders.

In this article, we report the micro-Raman spectroscopy study of blood sample from MI patients that can be used as a minimally invasive diagnostic technique for probing the disease by evaluating oxygenation status and the relative changes in phenylalanine and tyrosine concentrations. The Phe-Tyr ratio is an indicator about the presence of inflammation in case of MI patients. This approach requires only a minimal quantity of blood for evaluating the effect of medication and thus prognosis of the disease.

## Experimental methods

For the study, whole blood (1 to 2 ml) samples were collected from clinically confirmed acute MI patients (confirmed by ECG, Echocardiogram and/or troponin test) aged 35–70 years. First sample from the patient was collected at the time of admission to the hospital from the emergency triage and the second sample was taken 12–36 h after admission in the intensive coronary care unit (ICCU) after standard of care treatment. Patients involved in this study had comorbidities like diabetes and hypertension. Control blood samples were collected from asymptomatic healthy volunteers, aged 18–55 years, with no co-morbidities. Plasma is collected from whole blood sample by centrifuging at 5000 rpm for 5 min. First sample was labelled MI(BM) and second sample was labelled MI(AM). The blood samples labelled MI(BM) (myocardial infarction blood sample before medication) were collected from the Emergency Department, Kasturba Hospital, Manipal, and the MI blood samples labelled MI(AM) (myocardial infarction blood sample after medication) were collected from the ICCU, Kasturba Hospital, Manipal.

**Fig. 1:**
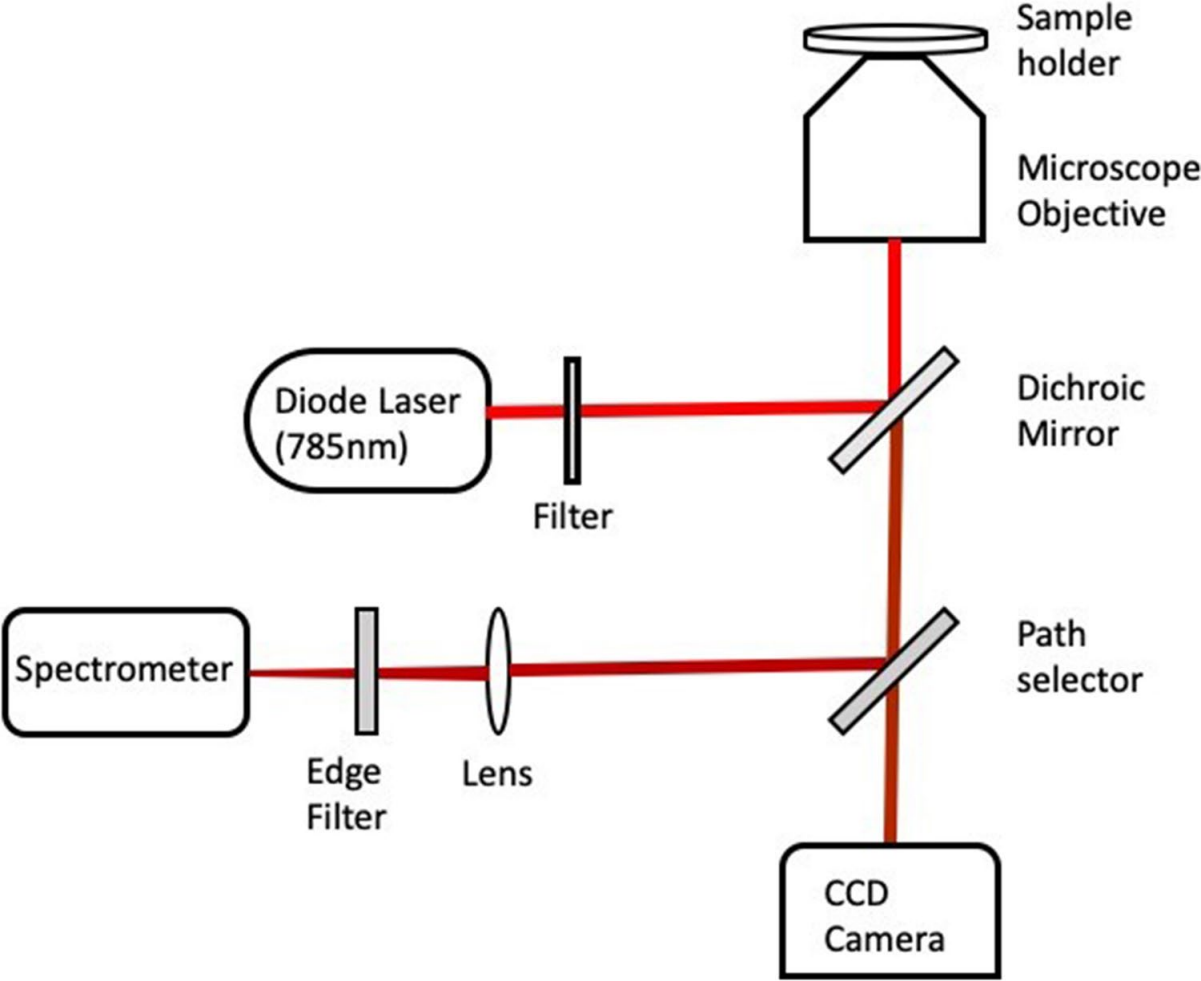
Schematic diagram of micro-Raman setup used for the experiments

Raman measurement of the blood samples has been carried out using the locally assembled micro-Raman instrument. The block diagram of the instrument is shown in Fig. 1 and more about the instrumentation will be available elsewhere [20]. Whole blood Raman spectra were recorded using ~ 15 mW Laser power, with 60 s exposure time and two accumulations. The Raman spectra of plasma samples were recorded using ~ 45 mW Laser power, with 60 s exposure time and two accumulations. In each category, 60 Raman spectra were recorded for the study from the two categories of samples. The origin pro 8 software was used for smoothing of Raman spectra using Savitzky–Golay method and baseline correction was carried out using Grams/A1 (Galactic Industries Corp) software. All the Raman spectra were unit vector normalized and principal component analysis (PCA) were carried out using Unscrambler X (version 10.4 CAMO, Norway) software.

## Result and discussion

### Raman spectral analysis of whole blood and plasma

**Fig. 2:**
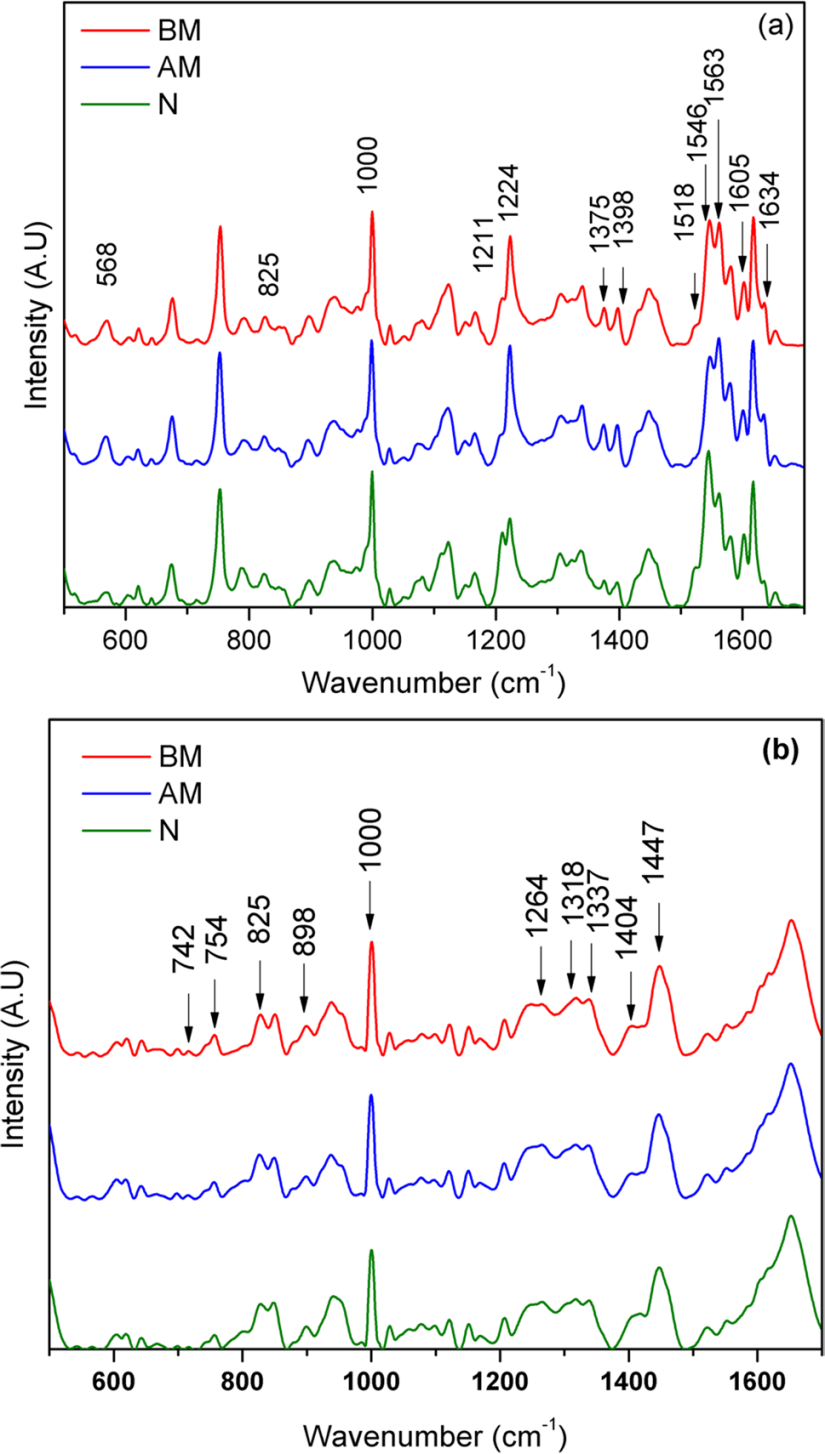
The comparison of averaged Raman spectra of whole blood (**a**) and plasma (**b**) of MI(BM), MI(AM) and Normal (N) samples

Blood samples are well suited for any disease diagnosis, due to its easy access, it transports oxygen, carbon dioxide, proteins, hormones, toxins, amino acids, metabolic products, nutrients etc. throughout the body and hence has the ability to reflect the biochemical changes accompanying with the progress of any disease [21]. Raman spectroscopic technique has the ability to provide spectral fingerprints of biomolecular changes during the occurrence of any disease which originated from the vibrational frequencies of molecular bonds within molecule/molecules. This technique has been of paramount importance in providing valuable insights to clinicians in disease diagnosis and prognosis of therapy [22, 23]. Averaged Raman spectra of whole blood (Fig. 2a) and plasma (Fig. 2b) collected from MI(BM) and MI(AM) patients and healthy volunteers are plotted in Fig. 2, where, each spectrum is an average of 60 spectra recorded from the different sample categories (MI(BM),MI(AM) and clinically Normal).

Significant spectral variations were observed in the Raman spectrum that correspond to oxygenation and deoxygenation characteristic markers of whole blood from MI patients with respect to control samples. Raman peaks at 1211 cm^−1^ and 1224 cm^−1^ arising from C-H methine deformation were regarded as the standard markers for hemoglobin oxygenation and deoxygenation states respectively. Another peak observed at 1634 cm^−1^ was also reported as a good representative of oxygenated hemoglobin. Similarly, spin marker region also holds interest in blood investigations due to the presence of oxy (1546 cm^−1^) and deoxyhemoglobin (1563 cm^−1^) Raman peaks as indicators. In addition, the hemoglobin oxygenation state can also be evaluated by monitoring the spectral signatures of pyrrole stretch frequencies (1375 cm^−1^ and 1398 cm^−1^) and the Fe-O_2_ stretch frequency(568 cm^−1^) [24, 25]. The assignments of the Raman peaks of the whole blood sample that had undergone changes are given in Table 1.

**Table 1:** Raman band assignments of whole blood samples

Raman band positions (cm^−1^)	Assignments
568	ν (Fe-O_2_)
825	γ_10_
1000	ν_45_, Phe
1211	ν_18_
1224	ν_42_
1375	ν_4_
1398	ν_20_
1518	ν_38_
1546	ν_11_
1563	ν_2_
1605	ν(C = = C)_venyl_
1634	ν_10_

ν—in plane mode; γ—out of plane mode.

The patients were admitted to the emergency department along with oxygen supplementation. The medical oxygen support has been continued during the treatment period when the patients were undergoing treatments in the ICU. Data given here for the normal samples were collected from healthy volunteers with 25–40 years of age group. It is clearly evident from Fig. 2 that all the oxygenation characteristic peaks were shown intensity enhancement in Raman spectra of MI patient in comparison with the control samples. Studies have shown that oxygen supply through face mask can elevate the oxygen level in the blood. As the viscosity of the blood decreases during the treatment, the resistance to blood flow reduces with overall effect of increased blood flow to and from the heart, resulting in an increased oxygen supply [26, 27]. In the present study, the MI samples have been collected from the patients who were already under external oxygen supply through facial mask as a part of the emergency treatment. This may be the causative factor for the increase in hemoglobin oxygenation found in case of MI blood samples.

The plasma Raman spectra of MI and Normal samples showed significant variations in terms of intensity of spectral band, which may be possibly due to changes in biological components in blood plasma. The assignments of the Raman peaks of the plasma sample showing changes are presented in Table 2. The increase in Raman band 898 cm^−1^ in MI sample is assigned to C–O–C starching. The Raman spectral band corresponding to 754 cm^−1^ and 1264 cm^−1^ corresponds to protein and amide III respectively, and is found to be increasing in case of MI to that of normal sample. The enhancement in the Raman peak at 1318 cm^−1^ (guanine) and at 1337 cm^−1^ (guanine and adenine) corresponding to DNA base pairs is observed in case of MI samples [28]. The increase in the concentration of cell free DNA in plasma is suggested to be a marker for MI. The cell free DNA originates as a result of cellular injury and it explains the degree of cell damage [29, 30]. The rise in Raman intensity of the peaks at 742 cm^−1^ and 1447 cm^−1^, corresponding to phospholipids is observed in MI samples. The decrease in intensity of Raman signal at 1404 cm^−1^ corresponding to glutathione is also seen in MI samples [31]. Glutathione is an important anti-oxidant and its role in oxygen radical scavenging thus preventing the cellular damage has been well established in earlier studies. The lower plasma level of glutathione, in various clinical conditions such as CVD, diabetes, arthritis and malignancies suggests the defensive role of glutathione against cellular damages [32].

**Table 2:** Raman band assignments of plasma samples

Raman band positions (cm^−1^)	Assignments
742	Phospholipids
754	Symmetric ring breathing of tryptophan
825	Out of plane ring breathing of tyrosine
898	C–O–C stretching
1000	C–C stretch, Phe
1264	C-N stretch
1318	CH_3_/CH_2_ wagging
1337	Ring mode
1404	Glutathione
1447	CH_3_CH_2_ bending vibration

The principal component analysis (PCA) was carried to visualize variation in MI and normal Raman plasma spectra. The Figure shows the Score 1 versus Score 2 plot of the plasma Raman spectra, where Score 1 covers 53.93% of variance and Score 2 having 63.74% of variance. The PCA results convey clear discrimination between the MI (BM) and MI (AM)) and Normal samples. The PCA results consolidate, relative changes observed in the Raman spectra of two categories of samples.

### Evaluation of phenylalanine and tyrosine Raman bands

**Fig. 3:**
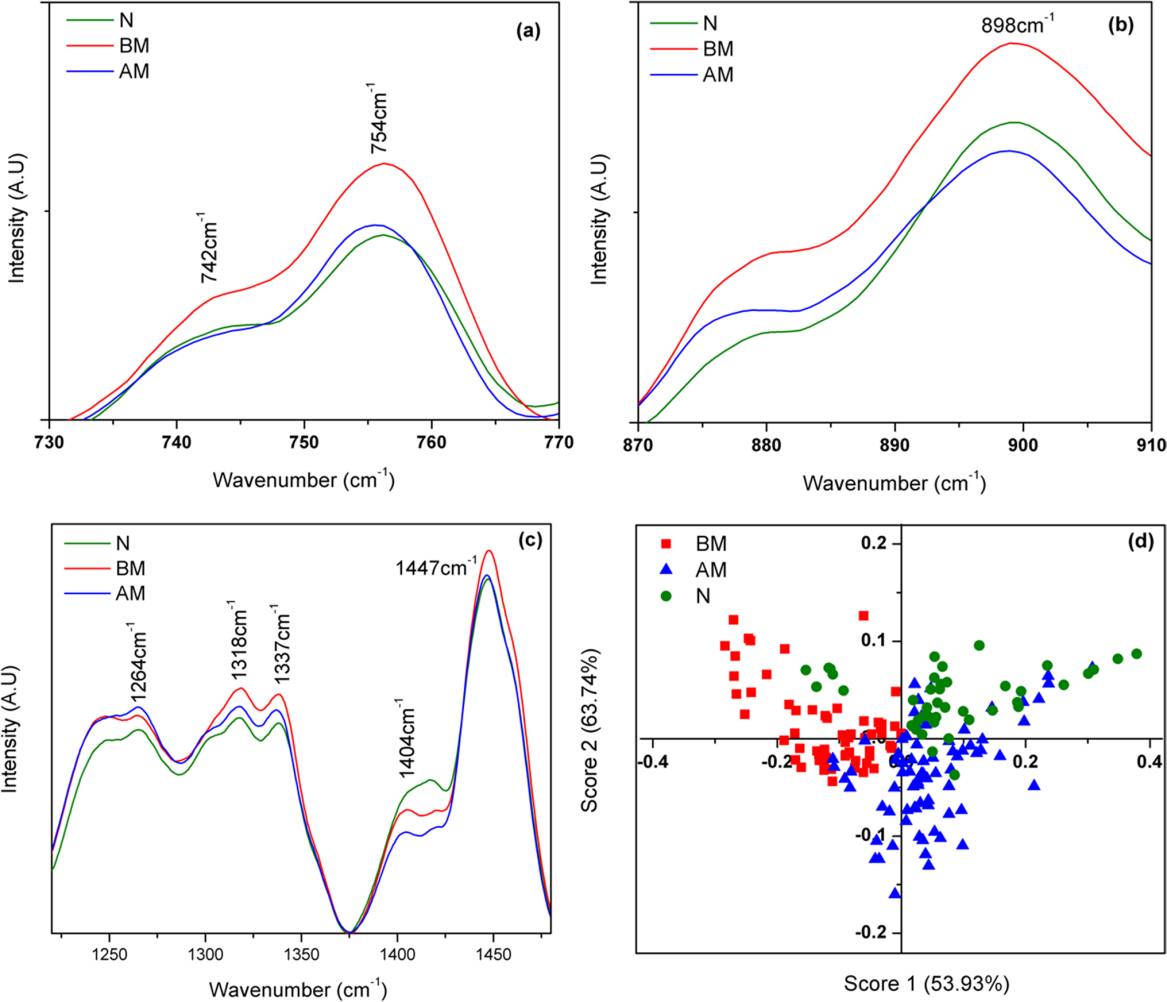
The average Raman spectra of Normal and MI (BM) and MI (AM) samples in the region (**a**) 730–770 cm^−1^, (**b**) 870–910 cm^−1^, (**c**) 1220–1480 cm.^−1^, and (**d**) PCA result of Raman spectra of MI (BM and AM) and normal samples (N)

Phe-Tyr ratio has been regarded as a vital indicator for inflammatory marker and immune activation in clinically abnormal patients. The inflammation and immune activation hinders the effective hydroxylation of phenylalanine to tyrosine in individuals suffering from cardiac disorders. In view of this, intensity of Raman peaks at 825 cm^−1^ and 1000 cm^−1^ that corresponds to tyrosine and phenylalanine respectively has been estimated. The Phe-Tyr ratio, peak intensities obtained for normal individuals and MI patients are given in Table 3 and Table 4. The bar plots obtained by calculating Phe-Tyr ratio (1000 cm^−1^/825 cm^−1^) for normal, MI (BM) and MI (AM) patients from the Raman spectra of blood and plasma are shown in Fig. 3a and b. As evident from the figure, the MI patients exhibited an increase in Phe-Tyr ratio with respect to healthy controls. In addition, the MI (AM) group has shown a decrease in this ratio as compared to MI (BM) samples. As mentioned earlier, previous literatures have linked higher Phe-Tyr ratio with the immune activation marker neopterin and C-reactive protein (CRP). Higher neopterin concentration in blood serum is correlated with an increased mortality rate in cardiovascular disease patients [33, 34]. The higher Phe-Tyr ratio indicates abnormal PAH enzyme activity, which is responsible for hydroxylation of phenylalanine to tyrosine. The PAH enzymatic activity is affected due to the deficiency in BH4 cofactor [13, 14]. The higher Phe-Tyr ratio observed in case of BM samples in whole blood and plasma indicates the scarcity of BH4 availability. The lower BH4 availability not only effects the PAH activity but also other codependent process namely, glyceryl ether monooxygenase and nitric oxide synthases (NOS) [35–37].

**Table 3:** Phe-Tyr ratio obtained from the Raman spectra of whole blood of MI (BM), MI (AM) and Normal samples

Sample MI(BM)	1000 cm^−1^/ 825 cm^−1^	Sample MI(AM)	1000 cm^−1^/ 825 cm^−1^	Sample Normal (N)	1000 cm^−1^/ 825 cm^−1^
BM1	4.19	AM1	4.22	N1	3.36
BM2	4.61	AM2	4.06	N2	4.06
BM3	4.92	AM3	4.57	N3	3.82
BM4	4.91	AM4	3.88	N4	3.62
BM5	5.38	AM5	4.2	N5	3.81
BM6	4.28	AM6	4.39	N6	3.73
BM7	5.04	AM7	4.18	N7	3.28
BM8	4.51	AM8	4.79	N8	3.76
BM9	4.81	AM9	3.35	N9	3.89
BM10	5.13	AM10	4.3	N10	3.53

**Table 4:** Phe-Tyr ratio obtained from the Raman spectra of blood plasma of MI (BM and AM) and Normal samples

Sample MI (BM)	1000 cm^−1^/ 825 cm^−1^	Sample MI(AM)	1000 cm^−1^/ 825 cm^−1^	Sample Normal (N)	1000 cm^−1^/ 825 cm^−1^
BM1	3.04	AM1	2.55	N1	2.05
BM2	2.69	AM2	2.28	N2	2.42
BM3	3.24	AM3	2.27	N3	2.28
BM4	2.86	AM4	3.12	N4	1.97
BM5	3.04	AM5	2.93	N5	2.63
BM6	2.6	AM6	1.94	N6	1.68
BM7	2.75	AM7	2.36	N7	2.1
BM8	2.78	AM8	2.6	N8	2.68
BM9	3.14	AM9	2.54	N9	1.88
BM10	3.39	AM10	2.45	N10	1.98

**Table 5:** Match/No-match results for whole Raman spectra tested against the MI (BM) calibration set

Sample number	Sample	Match	Mahalanobis (M)—distance	Spectral residual
1–17	BM, Whole blood	Yes	1.70–3.96	0.007–9.69
18–20	BM, Whole blood	No	4.16–6.85	1.24–1.84
21–39	N, Whole blood	No	4.00–22.80	0.02–5.23
40	N, Whole blood	Yes	2.23	7.43
1–17	BM, Plasma	Yes	0.52–2.65	2.51–9.53
18–20	BM, Plasma	No	3.85–5.51	0.01–1.46
21–40	N, Plasma	No	3.03–23.02	0.01–6.24

**Fig. 4:**
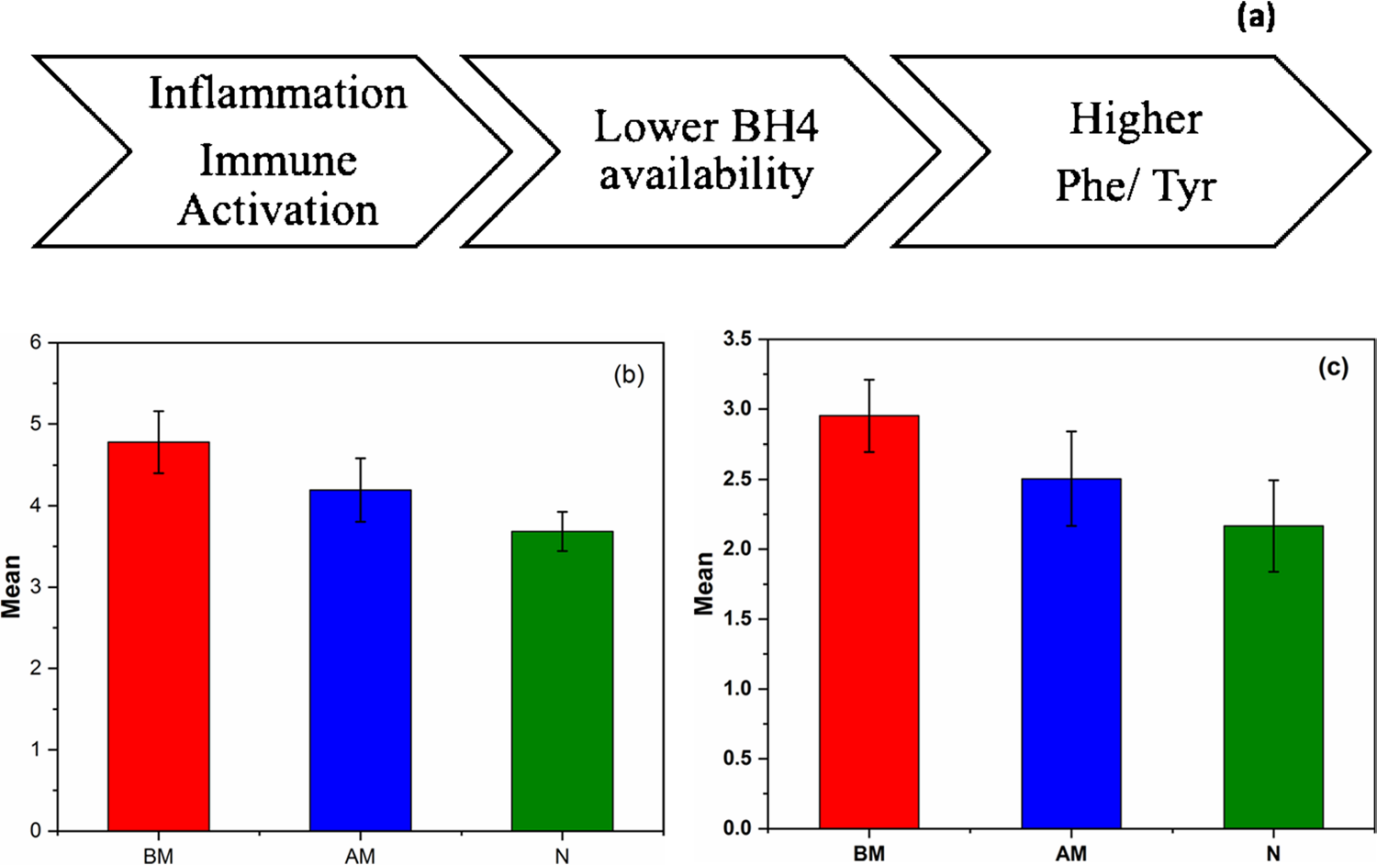
**a** Pictorial representation of Phe-Tyr ratio with inflammation and immune activation. The bar plot showing ratios of Raman peak intensities corresponding to phenylalanine (1000 cm^−1^) and tyrosine (825 cm^−1^) of MI (BM), MI (AM) and Normal samples from (**b**) Whole blood and (**c**) Blood plasma.

The pictorial representation showing the correlation between the Phe-Tyr ratio with inflammation and immune activation is illustrated in Fig. 4. The preliminary step in the production of BH4 is through enzyme GTP-cyclohydrolase I (GCH). The stimulations of proinflammatory stimuli’s, for instance cytokine tumor necrosis factor α (TNF—α) and lipopolysaccharide (LPS) on GCH causes the production of immune activation marker, neopterin in place of BH4. Literatures have also reported that the anti-inflammatory drugs such as salicylic acid, aspirin etc. suppress the neopterin level, thus altering the BH4 availability [38–40]. This may be the reason for the decrease in Phe/Tyr ratio observed in AM patients with respect to the BM sample. In brief, the increase in Phe-Tyr ratio observed in case of BM samples depicts possible inflammatory and immune activation process in myocardial infarction (MI). Similarly, the reduction in inflammation due to medication to patients may be the probable reason behind the decrease in the ratio.

**Fig. 5:**
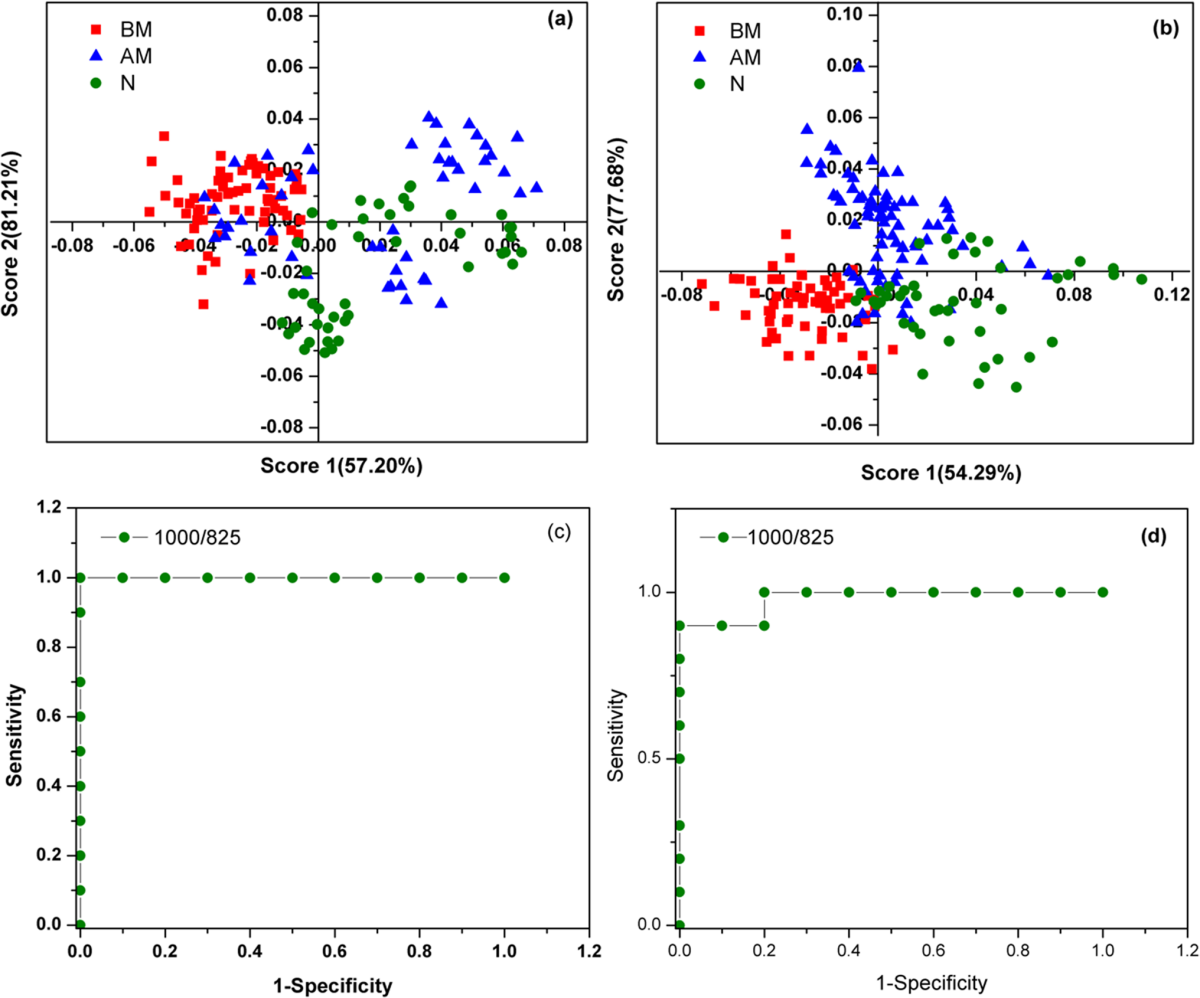
The PCA result of Raman spectra of MI(BM), MI(AM) and Normal samples (N): (**a**) Whole blood and (**b**) Blood plasma. The ROC curve for (**c**) Whole blood and (**d**) Blood plasma

The principal component analysis (PCA) was also performed to visualize the classification of the three groups of samples (Normal, MI (BM) and MI (AM)) as different by selecting the Raman spectral region corresponding to tyrosine (810–840 cm^−1^) and phenylalanine (990–1020 cm^−1^) using Unscrambler X software. Figure 5 shows the Score of Factor 1 versus Score of Factor 2 for whole blood (Fig. 5a) and plasma (Fig. 5b) samples. The scatter plot shows fairly good discrimination between MI (BM) samples and Normal samples with minimal overlap. Whereas significant overlap of samples was observed between MI (AM) samples and Normal samples. The observation strongly supports the earlier findings that there exists significant variation in phenylalanine and tyrosine concentrations in all the three categories of samples along with the tendency of mixing of MI(AM) samples with Normal resulting from the reduction in the inflammatory condition (lower Phe-Tyr ratio) while the MI patient is undergoing treatment.

The receiver operating characteristic (ROC) curve is plotted to evaluate the success of the diagnostic method. It gives the relation between sensitivity and specificity of a particular diagnostic test and also the optimum threshold value [41, 42]. Figure 5c and d shows ROC curve for whole blood and plasma samples obtained by considering Raman intensity ratio of phenylalanine to tyrosine. The optimum threshold value of Phe-Tyr ratio for whole blood and plasma samples are found to be 4.19 and 2.6 respectively. The area under the curve (AOC) for the ROC for whole blood and plasma samples is observed to be 100% and 98% respectively. The ROC curve for whole blood and plasma shows good diagnosis outcome indicating the success of selecting Raman intensity ratio of peaks corresponding to phenylalanine and tyrosine as a measure of inflammatory condition. The ROC curve also predict reliability of method in clinical use and the result obtained highlights the capability of the Raman spectroscopy analysis of whole blood and plasma in the diagnosis of MI.

Whole blood and plasma Raman spectra were subjected to Match/No match test. Procedure for Match/No match test involves a calibration set. In this case, the calibration set consists of thirty clinically certified MI sample Raman spectra. The spectra of the calibration set is then subjected to PCA to determine the parameters which are highly specific to the MI sample Raman spectra. For Match/ No match test, three parameters were considered namely factors, spectral residuals and M-distances. In PCA, initial factors cover maximum variation, in this case factor 3 gave good results for whole blood and plasma samples. The sum of difference between original spectrum and predicted spectrum from factors gives spectral residual [43]. The distance between the test sample point and the mean of all the other points in the class is determined as the M- distance, where distance is measured in terms of standard deviation in all dimensions for range of variation in a class.

M- distance matrix is given by,

$$\mathrm M=\left(\mathrm S^\prime\mathrm S/\left(\mathrm n-1\right)\right)$$

M- distance, D for any test sample is given by

$$\mathrm D^2=\mathrm{SM}^{-1}\mathrm S^{'}$$

M is an f x f M- distance matrix and S is an n x f matrix of calibration set sample’s PCA scores, where n & f are number of samples and PCA scores respectively [44]. The result of the Match/No match test is shown in Table 5. In whole blood and plasma samples up to sample number 17 showed Match with the MI calibration set, whereas three samples gave No-Match leading to false result. Similarly, in normal samples, samples up to 19 had No Match with MI calibration set, but one sample Matched with MI calibration set in whole blood sample, whereas in plasma sample, all twenty samples had No Match with MI calibration set. The Match/No Match test results showed the same 85% sensitivity for whole blood and plasma samples, while, specificity is observed to be 95% and 100% for whole blood and plasma samples, respectively.

Direct detection of BH4 in blood sample demands complex preanalytical procedures which limits its use in clinical application [39, 45]. In view of this, the Phe-Tyr ratio can be used as an indirect marker of BH4 availability, since it regulates the PAH enzyme activity. This indicates the diagnostic importance of probing Phe-Tyr ratio to evaluate the inflammation process occurring in cardiac patients. This ratio can also be exploited for evaluating the effectiveness of anti-inflammatory drugs on subjects by acquiring Raman spectra of blood samples with minimal sample preparation. Prospective rigorous validation using more number of clinical samples is necessary to establish Raman-spectroscopy as a rapid and objective tool for inflammation monitoring in routine clinical usage.

## Conclusion

Despite being a highly sensitive and label free technique for biological and medical applications, Raman spectroscopy requires minimal sample preparation and low sample volume. The potential of this technique has not yet been explored for cardiovascular disease studies. The present study using micro-Raman spectroscopy is an attempt to explore the proinflammatory and immune activation process in MI patients. Raman spectral signatures have clearly shown an elevated Phe-Tyr ratio in MI patients with respect to normal. The Phe-Tyr ratio of patients with MI after medication displayed shifting of value from higher level to lower in case of medicated, indicating the positive response of medication for inflammatory condition. The PCA results showed good discrimination between patients’ sample without medication and normal samples confirming the variation in the relative concentration of phenylalanine and tyrosine molecules. The Match/No Match test results showed the same 85% sensitivity for whole blood and plasma samples, while, specificity is observed to be 95% and 100% for whole blood and plasma samples, respectively. Phe-Tyr ratio being a measure of PAH activity, in turn indicates the BH4 availability, which can be related to immune activation and inflammatory process present in MI patients. This study strongly support the diagnostic importance of Phe-Tyr ratio determination using Raman spectroscopy for MI samples.

## References

[1] Buttar HS, Li T, Ravi N (2005). Prevention of cardiovascular diseases: role of exercise, dietary interventions, obesity and smoking cessation. Exp Clin Cardiol.

[2] World Health Organization (2013) Health topics: cardiovascular diseases. http://www.Who.Int/Topics/Cardiovascular_diseases/En/. Accessed June 2021

[3] Boersma E, Mercado N, Poldermans D (2003). Acute myocardial infarction. Lancet.

[4] Thygesen K, Alpert JS, Jaffe AS (2012). Third universal definition of myocardial infarction. J Am Coll Cardiol.

[5] Bosshart F, Utzinger U, Hess OM (1992). Fluorescence spectroscopy for identification of atherosclerotic tissue. Cardiovasc Res.

[6] Frangogiannis NG, Smith CW, Entman ML (2002). The inflammatory response in myocardial infarction. Cardiovasc Res.

[7] Reed GW, Rossi JE, Cannon CP (2016). Acute myocardial infarction. Lancet.

[8] Thygesen K, Jaffe AS, Chaitman BR (2018). Fourth universal definition of myocardial infarction (2018). J Am Coll Cardiol.

[9] Fang L, Moore X, Dart AM, Wang L (2015). Systemic inflammatory response following acute myocardial infarction. J Geriatr Cardiol.

[10] Dutta P, Courties G, Wei Y (2012). Myocardial infarction accelerates atherosclerosis. Nature.

[11] Melamed KH, Goldhaber SZ (2014). Inflammation and myocardial infarction. Circulation.

[12] Münzel T, Daiber A (2018). Role of endothelial and macrophage tetrahydrobiopterin in development and progression of atherosclerosis: BH4 puzzle solved?. Cardiovasc Res.

[13] Kopple JD (2007). Phenylalanine and tyrosine metabolism in chronic kidney failure. J Nutr.

[14] Matthews DE (2007). An overview of phenylalanine and tyrosine kinetics in humans. J Nutr.

[15] Eberhardt K, Stiebing C, Matthaüs C, Schmitt M, Popp J (2015). Advantages and limitations of Raman spectroscopy for molecular diagnostics: an update. Expert Rev Mol Diagn.

[16] Cialla-May D, Zheng XS, Weber K, Popp J (2017). Recent progress in surface-enhanced Raman spectroscopy for biological and biomedical applications: from cells to clinics. Chem Soc Rev.

[17] Enejder AMK, Koo T-W, Oh J, Hunter M, Sasic S, Feld MS, Horowitz GL (2002). Blood analysis by Raman spectroscopy. Opt Lett.

[18] Jafarzadeh F (2018). Graphene based coating on baremetal stents improves human coronary artery endothelial cell growth. Cardiovasc Res.

[19] Amodio G, Antonelli G, Francesca DS (2010). Cardiac biomarkers in acute coronary syndromes. Curr Vasc Pharmacol.

[20] Bankapur A, Zachariah E, Chidangil S, Valiathan M, Mathur D (2010). Raman tweezers spectroscopy of live, single red and white blood cells. PLoS One.

[21] Wang H, Zhang S, Wan L, Sun H, Tan J, Su Q (2018). Screening and staging for non-small cell lung cancer by serum laser Raman spectroscopy. Spectrochim Acta A Mol Biomol Spectrosc.

[22] Xue L, Yan B, Li Y, Tan Y, Luo X, Wang M (2018). Surface-enhanced raman spectroscopy of blood serum based on gold nanoparticles for tumor stages detection and histologic grades classification of oral squamous cell carcinoma. Int J Nanomed.

[23] Bai Y, Yu Z, Yi S, Yan Y, Huang Z, Qiu L (2020) Raman spectroscopy-based biomarker screening by studying the fingerprint characteristics of chronic lymphocytic leukemia and diffuse large B-cell lymphoma. J Pharm Biomed Anal 190. 10.1016/j.jpba.2020.113514 10.1016/j.jpba.2020.113514 32827998

[24] Sato H, Chiba H, Tashiro H, Ozaki Y (2001). Excitation wavelength-dependent changes in Raman spectra of whole blood and hemoglobin: comparison of the spectra with 514.5-, 720-, and 1064-nm excitation. J Biomed Opt.

[25] Ohira S, Tanaka H, Harada Y (2017). Label-free detection of myocardial ischaemia in the perfused rat heart by spontaneous Raman spectroscopy. Sci Rep.

[26] McLellan SA, Walsh TS (2004). Oxygen delivery and haemoglobin. Contin Educ Anaesthes Crit Care Pain.

[27] Amini A, Mahdavipour A (2019) The effect of oxygen therapy on oxidative stress index in patients with acute myocardial infarction; a letter to the editor. Arch Acad Emerg Med 7. 10.22037/aaem.v7i1.2 PMC6377221 30847437

[28] Rekha P, Aruna P, Bharanidharan G (2015). Near infrared Raman spectroscopic characterization of blood plasma of normal, oral premalignant and malignant conditions - a pilot study. J Raman Spectrosc.

[29] Xie J, Yang J, Hu P (2018). Correlations of circulating cell-free DNA with clinical manifestations in acute myocardial infarction. Am J Med Sci.

[30] Polina IA, Ilatovskaya DV, DeLeon-Pennell KY (2020). Cell free DNA as a diagnostic and prognostic marker for cardiovascular diseases. Clin Chim Acta.

[31] Vargas-Obieta E, Martínez-Espinosa JC, Martínez-Zerega BE (2016). Breast cancer detection based on serum sample surface enhanced Raman spectroscopy. Lasers Med Sci.

[32] Shimizu H, Kiyohara Y, Kato I (2004). Relationship between plasma glutathione levels and cardiovascular disease in a defined population: the Hisayama study. Stroke.

[33] Mangge H, Schnedl WJ, Schröcksnadel S (2013). Immune activation and inflammation in patients with cardiovascular disease are associated with elevated phenylalanine-to-tyrosine ratios. Pteridines.

[34] Wannemacher RW, Klainer AS, Dinterman RE, Beisel WR (1976). The significance and mechanism of an increased serum phenylalanine tyrosine ratio during infection. Am J Clin Nutr.

[35] Cai S, Khoo J, Channon KM (2005). Augmented BH4 by gene transfer restores nitric oxide synthase function in hyperglycemic human endothelial cells. Cardiovasc Res.

[36] Pastor CM, Williams D, Yoneyama T (1996). Competition for tetrahydrobiopterin between phenylalanine hydroxylase and nitric oxide synthase in rat liver. J Biol Chem.

[37] Higgins CE, Gross SS (2010) Tetrahydrobiopterin. An essential cofactor for nitric oxide synthases and amino acid hydroxylases. In: Louise J. Ignarro (ed), Nitric Oxide, 2nd edn. Academic Press, Cambridge, pp 169–209. 10.1016/B978-0-12-373866-0.00006-X

[38] Worthley MI, Kanani RS, Sun YH (2007). Effects of tetrahydrobiopterin on coronary vascular reactivity in atherosclerotic human coronary arteries. Cardiovasc Res.

[39] Geisler S, Gostner JM, Becker K (2013). Immune activation and inflammation increase the plasma phenylalanine-to- tyrosine ratio. Pteridines.

[40] Bendall JK, Douglas G, McNeill E (2014). Tetrahydrobiopterin in cardiovascular health and disease. Antioxid Redox Signal.

[41] Bhat S, Patil A, Rai L et al (2012) Application of HPLC combined with laser induced fluorescence for protein profile analysis oftissue homogenates in cervical cancer. Sci World J 2012. 10.1100/2012/976421 10.1100/2012/976421 PMC3356758 22645492

[42] Patil A, Prabhu V, Choudhari K.S. , Unnikrishnan V.K., George S.D, Ongole R, Pai K.M, Shetty. J.K, Bhat S, Kartha V.B, Chidangil S (2010) Evaluation of high-performance liquid chromatography laser-induced fluorescence for serum protein profiling for early diagnosis of oral cancer. J Biomed Opt 15. 10.1117/1.3523372 10.1117/1.3523372 21198211

[43] Bhat S, Patil A, Rai L, Kartha VB, Santhosh C (2010). Protein profile analysis of cellular samples from the cervix for the objective diagnosis of cervical cancer using HPLC-LIF. J Chromatogr B.

[44] Sujatha, Lavanya R, Mahato KK (2015) Serum protein profile study of normal and cervical cancer subjects by high performance liquid chromatography with laser-induced fluorescence. J Biomed Opt 13. 10.1117/1.2992166 10.1117/1.2992166 19149028

[45] Mcneill E, Channon KM (2017). The role of tetrahydrobiopterin in inflammation and cardiovascular disease. Thromb Haemost.

